# Editorial: Role of Blood Cells in Inflammatory and Vascular Disorders

**DOI:** 10.3389/fphar.2020.585705

**Published:** 2020-09-11

**Authors:** Vincenzo Brancaleone, Jesmond Dalli, Roberta d’Emmanuele di Villa Bianca, Simona Pace

**Affiliations:** ^1^ Department of Science, University of Basilicata, Potenza, Italy; ^2^ William Harvey Research Institute, Barts and The London School of Medicine and Dentistry, Queen Mary University of London, London, United Kingdom; ^3^ Department of Pharmacy, University of Naples Federico II, Naples, Italy; ^4^ Institute of Pharmacy, Friedrich Schiller University Jena, Jena, Germany

**Keywords:** lipid mediator, inflammation, resolution, vascular disease, leukocyte

The vascular endothelium is regulated by a range of mediators produced by circulating blood cells. This interplay is crucial to control vascular function together with inflammatory processes. Indeed, injury or infections generate a cascade of events that stimulate leukocytes transmigration leading to release of both pro- and antinflammatory molecules. One of the key steps in the pro-resolutive response is associated with the limitation of neutrophil emigration into inflamed tissues, together with an increase in their clearance. One of the pivotal players in this process is represented by galectin 1 (Gal-1), which can strongly inhibit neutrophil transmigration as well as facilitate neutrophils removal (Law et al.) (**Figure 1**). In addition, Gal-1 is also able to modulate the number of macrophages and levels of pro- and anti-inflammatory cytokines, thus controlling the outcome of the inflammatory process, also involving IFN-β in this mechanism (Yaseen et al.). Extracellular vesicles (EV) also play a central role in tempering vascular responses. These membrane-bound vesicles that facilitate are released by different cell types, and their content reflects both the cell of origin and its activation status. Recent studies also demonstrate that EV derived from platelets, leukocytes, and endothelial cells control the interplay and interactions between leukocytes and platelets and between platelets and endothelial cells (Oggero et al.) (**Figure 1**). Among circulating blood cells, platelets have a peculiar role in vascular homeostasis and thrombosis and are an important source of purine nucleotides and nucleosides. The imbalance of extracellular purine levels is associated with an increase of cardiovascular risk, and the role of CD39 might be crucial to predict platelets’ reactivity and their ability to control nucleotides’/nucleosides’ effects (Caiazzo et al.) (**Figure 1**). Interestingly, cardiovascular diseases are strictly linked to several chronic inflammatory pathologies, including asthma. This association relies on the actions of inflammatory mediators such as IL-4, IL-6, IL-9, IL-17A, IL-33, and TNF-α that somehow affect endothelial function at different vascular levels, thus generating vascular dysfunction leading to atherosclerosis (Gurgone et al.). Although there is a growing body of evidence that highlights the importance of interactions between blood cells and endothelium, there is still a need to further elucidate the mechanisms involved in terms of cell-cell activation in the view of a possible therapeutic approach tackling inflammatory-based cardiovascular diseases (IBCDs). Interestingly, apart from different molecules undergoing preclinical and clinical studies, natural products could also provide an interesting source of therapeutic items. Indeed, molecules like ginkgolic acid (GA) could potentially target multiple key enzymes involved in the biosynthesis of pro-inflammatory mediators (Gerstmeier et al.) (**Figure 1**). Therefore, a multi-target approach could limit the inflammatory response at different levels, including vasculature and heart. These two organs represent the main targets in several pathologies, including diabetes, where cardiovascular complications represent the main cause of death in diabetic patients. Intriguingly, the use of isolated red blood cells (RBC) from diabetic patients with a different degree of glycemic control seems to have an effect on cardiovascular function in terms of post-ischemic recovery, possibly associated to RBC-arginase (Mahdi et al.).

**Figure 1:**
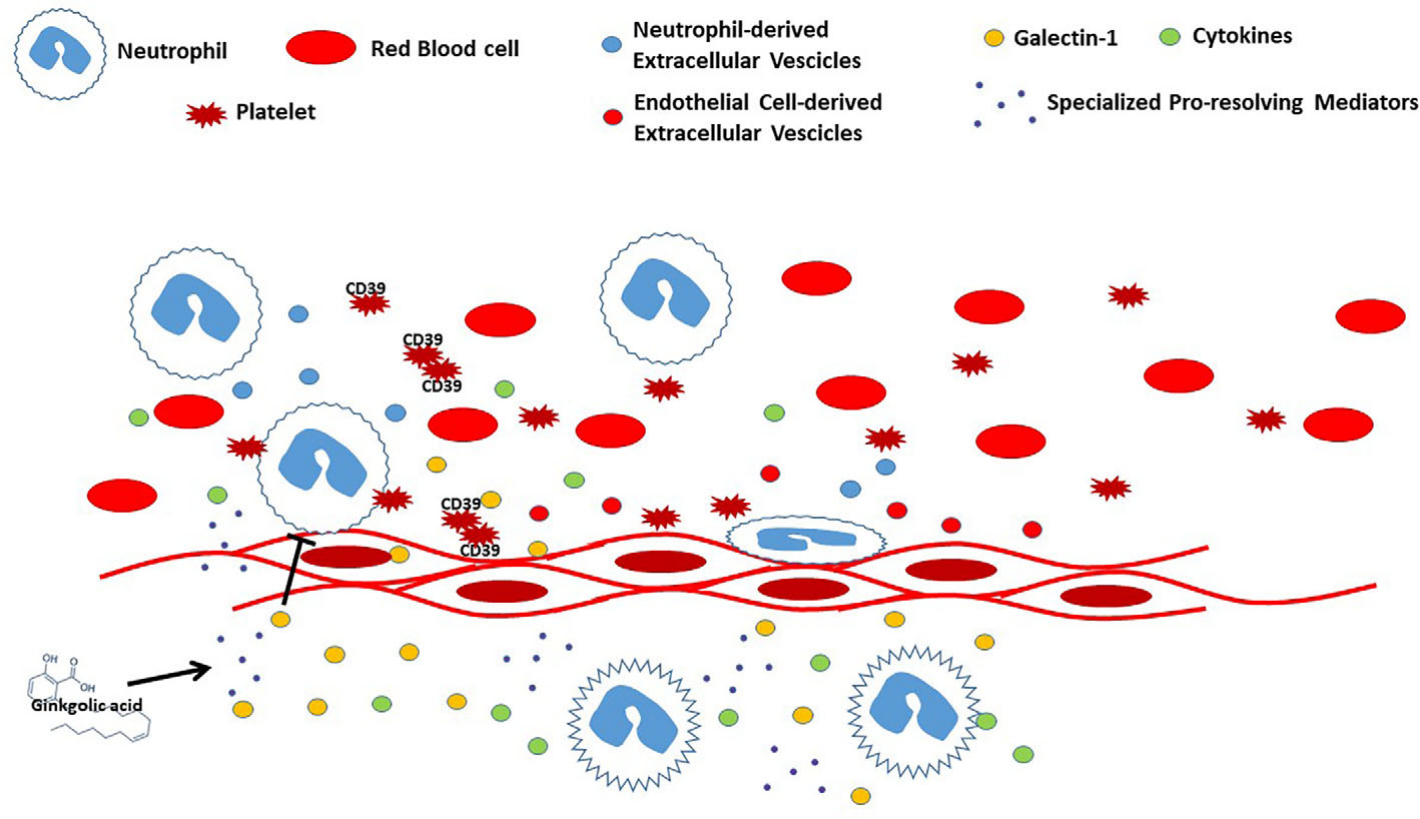
Representation of interplay between blood cells and endothelium during vascular inflammation.

All findings reported in this collection contribute to shed new light on the pathophysiological events driving vascular and inflammatory diseases, events that occur at the interface between blood cells and endothelium. We trust that this Research Topic will underscore the need for furthering our understanding in these fundamental mechanisms of disease, further stimulating the interest of scientists working in diverse disciplines.

## Author Contributions

VB wrote the draft of the editorial and submitted the final version. JD, RD’E, and SP revised the draft.

## Conflict of Interest

The authors declare that the research was conducted in the absence of any commercial or financial relationships that could be construed as a potential conflict of interest.

